# Serum 25-Hydroxyvitamin D Concentrations and Indicators of Mental Health: An Analysis of the Canadian Health Measures Survey

**DOI:** 10.3390/nu9101116

**Published:** 2017-10-13

**Authors:** Filmer Chu, Arto Ohinmaa, Scott Klarenbach, Zing-Wae Wong, Paul Veugelers

**Affiliations:** 1 Department of Medicine, Faculty of Medicine and Dentistry, University of Alberta, Edmonton, AB T6G 2G3 Canada; swk@ualberta.ca (S.K.); zingwae@ualberta.ca (Z.W.); 2 School of Public Health, University of Alberta, Edmonton, AB T6G 1C9, Canada; aohinmaa@ualberta.ca (A.O.); paul.veugelers@ualberta.ca (P.V.)

**Keywords:** vitamin D, Canada, depression, mental health

## Abstract

The main function of vitamin D is calcium homeostasis. However, emerging evidence has correlated adequate serum 25-hydroxyvitamin D (25(OH)D) concentrations with better mental health. The objective of this study is to investigate the association of serum 25(OH)D concentrations with indicators of mental health such as depression, anxiety, and stress. Associations of serum 25(OH)D concentrations with four indicators of mental health were examined using ordered logistic regression models with increasing specificity that account for demographics, socio-economic status, and health. Margin effects are used to determine the probability of the average adult Canadian being in the best mental health state by groupings of serum 25(OH)D concentrations. A robust association between serum 25(OH)D concentrations and the indicators of mental health were observed. In the fully adjusted ordered logistic model, an average Canadian appeared more likely to experience better mental health when serum 25(OH)D concentrations were higher. This study adds to the weight of the existence of an association between vitamin D status and mental health, but, as this study is cross sectional, it does not establish causality. Due to the low risk of harm from toxicity and the relative modest costs of vitamin D supplements, more research to establish the effectiveness and causality of this relationship is recommended.

## 1. Introduction

Mental health may be impacted by a range of mood disorders that effect both thinking and behavior such as depression, anxiety, and schizophrenia, as well as in substance use disorder. One in every five Canadians experiences a mental health problem, and by the time Canadians reach 40 years of age, one in every two Canadians have or have had a mental illness [1]. Reducing this disease burden will have a cascading effect on the health care system, from reduce direct cost on mental health professionals to the indirect cost of reduced substance abuse [2]. This study focuses on three indicators of mental health: depression, anxiety, and stress. The notion that relative inexpensive vitamin D supplements can improve mental health outcomes warrants an exploratory Canadian study. Despite vitamin D fortification, Canadians are not getting enough vitamin D to benefit from the potential protective effects especially during the winter [3].

The main function of vitamin D is to regulate the absorption (homeostasis) of calcium for better bone health [4,5]. However, emerging evidence has correlated adequate levels of serum 25-hydroxyvitamin D (25(OH)D) concentrations with better scores on indicators of mental health [6]. Active vitamin D binds to vitamin D receptors (VDR) to regulate physiologic functions of the body such as emotional well-being and stress. VDRs are found in more than 30 cell types throughout the body [7], including neuronal and glial cells [8] in the cortex and hippocampus, which have been implicated in the pathophysiology of mood [9]. Experiments on rodents have demonstrated alterations to brain function and behavior due to vitamin D deficiency [10]. Active vitamin D also regulates tyrosine hydroxylase, which in turn regulates the production of the mood regulating neurotransmitters, norepinephrine and dopamine [11]. The absolute or relative lack of norepinephrine is associated with most, if not all, types of mental health outcomes [11]. Lower dopamine levels are associated with mental health issues such as diminished motivation and psychomotor retardation [12]. Vitamin D may indirectly regulate mood by stimulating genes that produce neurotransmitters that relieve depressive emotions [13]. This biological link between vitamin D and mood raises the hypothesis of whether adequate vitamin D levels are associated with reduced probability of mental health illness.

Previous studies have found evidence that low serum 25(OH)D concentrations are associated with depression [14]. This includes studies from various countries showing a positive relationship between serum 25(OH)D concentrations and mental health indicators [15,16] and two community based samples of older Canadians [16,17]. It has been suggested that over one third of the Canadian population have suboptimal serum 25(OH)D concentrations [15]. If a causal relationship exists, this may be of considerable importance given the high rate of mental health illness in Canada [1]. This study investigates the relationship between serum 25(OH)D concentrations and mental health indicators using an established survey among a large sample that is representative of Canadians. 

## 2. Methodology and Dataset

The Canadian Health Measures Survey (CHMS) is a cross-sectional survey conducted every two years, developed and conducted by Statistics Canada in partnership with Health Canada and the Public Health Agency of Canada. Currently, three cycles are available from year 2007 to 2013. The CHMS covers the population aged 3 to 79 years living in the ten provinces. The data excludes people who are living in the three territories, living on reserves and other Aboriginal settlements, full-time members of the Canadian Forces, part of the institutionalized population, and residents of certain remote regions. Altogether, these exclusions represent approximately 4% of the Canadian population [18]. This study will include Canadians over the age of 18 and non-pregnant (sample size 7518). Sixteen mobile examination centers across Canada with trained professional collected blood samples for the assessment of serum 25(OH)D concentrations (expressed in nmol/L) constituting the exposure of interest. 

The gold standard assessment of mental health and well-being is an assessment from a mental health professional. However, this is unfeasible, impractical, and costly to obtain for a nationally representative dataset. This study uses four proxy measures of depression, anxiety, and stress. The first proxy exists in two cycles of the CHMS contains questions designed to derive the various components of the Health Utility Index 3 (HUI3). The HUI3 was developed in Canada and was designed to quantify overall health using eight attributes measured on a scale between one and six, where one indicated a better health state. The emotional attribute is extracted as one of proxies of depression/anxiety and was determined by the following question “Would you describe yourself as being usually: happy and interested in life; somewhat happy; somewhat unhappy; unhappy with little interest in life; so unhappy that life is not worthwhile.” The remaining three proxies are available in all three cycles of the CHMS. The second proxy is self-perceived mental health which, was measured by the question “In general, would you say your mental health is: Excellent; very good; good; fair; poor.” The remaining two proxies are self-perceived stress and self-perceived general health. Stress is measured by asking the respondent “Thinking about the amount of stress in your life, would you say that most days are: not at all stressful; not very stressful; a bit stressful; quite a bit stressful; extremely stressful.” The final proxy is a measure of general health but it can be argued that the belief of one’s general health is associated with their mental health and as such this measure will be used as a tertiary proxy of depression, anxiety, and stress. General health is measured by asking the respondent “in general, would you say your health is: Excellent; very good; good; fair; poor.” All four proxies are modelled with the initial two cycles stacked and resampled with the bootstrap weights provided by Statistics Canada to increase the statistical power and to provide a representative sample of the Canada Adult population. Proxies with three cycles are independently restacked and resampled with the appropriate bootstrap weights as a robustness check. 

The relationship between vitamin D status and indicators mental health is entangled by many observable confounders including demographics, socio-economic status, chronic conditions, smoking and drinking, illicit drug use and labor force status. Demographic controls in this study include age, sex, marital status, education, and ethnicity. Socio-economic status is controlled with household income and if the respondent is a student. Lifestyle controls consist of smoking, drinking, drug use (both prescription and street drugs). Health conditions are controlled with binary indicators for chronic and acute conditions (based on self-reported official diagnostics). The specific variables are also described in Table 1. Ordered logistic regression analyses will be performed with increasing specificity to ensure robustness of the results. The dependent variables are the mental health proxies as described (Table 2). Each proxy will be used independently with increasing model specifications to ensure robustness of results. 

Unobservable confounders such as Seasonal Affect Disorder (SAD) are periodic major depression occurs in some people during late fall to early spring, which may affect the indicators of mental health. Some patients have the opposite occurrence: depressive symptoms during spring and summer. Regardless of the season, the depression episodes occur during the same seasons every year [19]. The proposed datasets accounts for seasonality effects by uniformly distributing the 16 mobile examination center collection sites by region between the collection years [18].

## 3. Results

The characteristics of the population (Table 1) indicate an average age for adult Canadians (Canadians over the age of 18, excludes all pregnant females) is 45 with an average household income of $77,550. The average weight and height are 78 kg and 169 cm, respectively. Approximately 50% of Canadians are married. There is an equal split between males and females. Approximately 25% of the population graduated with an university degree or higher. Approximately 17% of the population smokes daily, and 68% consume alcoholic beverages on a regular basis. Over 50% of the population is considered inactive. Approximately 42% of Canadians have a normal weight. The indicators of mental health (Table 2) indicate that approximately 78% of Canadians are happy in life, and 34% have excellent self-perceived mental health. The indicator of self-perceived stress seems quite normally distributed, with 44% of Canadians having “a bit” of self-perceived stress. Most Canadians have a self-perceived health of being in the good, very good, and excellent category. 

Emotional health has a positive association with serum 25(OH)D concentrations across all models (Table 3). For every 25 nmol/L increase of serum 25(OH)D concentrations, there is an average of a 1.16-unit increase in log odds (or a 76% increase of being in the best emotional health category of the HUI3 index, calculated by converting log odds to probability) across all models that controls for demographics, socioeconomic status, lifestyle choices, and health of the respondent. Similar increase in predictive probability of being in a better mental health state are found for self-perceived mental health (75%), self-perceived stress (75%), and self-perceived general health (76%) for every 25 nmol/L increase of serum 25(OH)D concentrations in the blood stream. 

**Figure 1:**
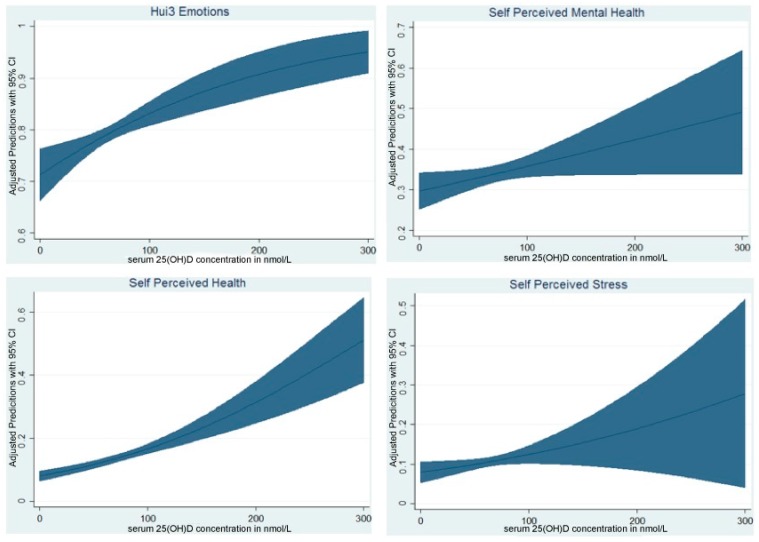
Adjusted probability with 95% confidence interval of being in the best mental health state by serum 25(OH)D concentrations.

The adjusted probability of being in the best health state for both the HUI3 emotional health category and self-perceived stress category increases with higher serum 25(OH)D concentrations. This probability is based on the average Canadian as defined previously. Similar adjusted probabilities of being in the best health state were found for self-perceived mental health and self-perceived general health. The probability of the average Canadian being in the best mental health state has an upward tendency as serum 25(OH)D concentrations increases and more so when moving from very low levels to 100 nmol/L. This is also when the confidence interval is narrowest suggesting a higher confidence in the prediction (Figure 1).

## 4. Robustness

All outcome variables of interest (self-perceived mental health, general health, and stress) available in Cycle 3 underwent the same regression models with all three cycles stacked with the appropriate Statistics Canada bootstrap weights. All results are consistent if not better compared with the main analysis. The confidence intervals are narrower when the three cycles are stacked. This is most likely due to the increase sample size that resulted in an increase of statistical power. All robustness results are available upon request from the corresponding author. 

## 5. Limitations

There are two important limitations to this study. The first is that this study can only determine an association and not a causal effect. In other words, it is unclear whether vitamin D produces better scores on the indicators of mental health or if lower depression and anxiety leads to higher serum 25(OH)D concentrations in the blood from better nutrition and/or outdoor activities. In other words, someone with depression and anxiety may not venture outside (less exposure to sunlight), and someone without depression and anxiety might be more active and go outside more. The authors attempted to estimate the casual affect through instrumental variable regression methods but were unable to find a valid instrument in the dataset, with the inability to control for all unobservable confounders such as nutrient-nutrient interactions and other individual heteroscedasticities.

The second major limitation is the method of ascertaining mental health. To accurately measure mental health states, a standard method completed by health professionals would be required. However, this is unrealistic, impractical, and costly for a nationally representative dataset. It is also very likely that due to the self-perceived nature of the questionnaires, these proxies are likely to be underestimated. Some of the proxies utilized in this study stem from health-related quality of life measurements, and we acknowledge that these may not be as good compared to mental health related quality of life instruments. Linking of other datasets has also been considered, but due to privacy rules with the CHMS dataset, this was not an option. 

## 6. Discussion

This study reveals robust positive associations between serum 25(OH)D concentrations and indicators of mental health (depression and anxiety) after controlling for demographics, socio-economic status, chronic conditions, smoking and drinking, illicit drug use, and labor force status. This study also found that higher serum 25(OH)D concentrations by each increment of 25 nmol/L increases the probability of the average Canadian to be in the best mental health and general health state by an average of 76%. 

Accurate measures of mental health and well-being ideally would include an assessment from a mental health professional which is unfeasible, impractical, and costly to obtain for a large nationally representative study sample. This study uses four established proxy measures of mental health. However, there are inherent biases, such as response bias due to the nature of the question. These biases must be weighed against the possible benefits in order to assess the potential overall benefit. 

Vitamin D is generally well tolerated, and adverse events and toxicity are rare when it is taken appropriately. To raise serum 25(OH)D concentrations to optimal amounts as shown in previous studies, Canadians would need a daily intake of 1000–4000 IU/day. However, Canadians on average can only obtain 200–300 IU/day from food sources [20] and live at high latitudes and thus have less sun exposure and subcutaneous vitamin D synthesis. Canadians would need to obtain the daily intake of vitamin D in arrays through supplements over a long period of time to see any benefits due to the short half-life of vitamin D (3 weeks).

Studies in countries such as China, England, Europe, Japan, The Netherlands, and United States have shown a robust relationship between vitamin D status and depression [6,15,16,21,22]. Two studies in a sample of older Canadians participating in a preventive health program showed that higher serum 25(OH)D concentrations were associated with improved health related quality of life [16,17]. Those observations, however, cannot be generalizable because they were in a selected group. The present study is the first to extend the finding to the entire Canadian adult population. Given the burden of mental health issues in Canada, and the large proportion of the Canadian population with suboptimal serum 25(OH)D concentrations [15], the present study adds to the support of the notion that relative inexpensive vitamin D supplements can prevent mental health problem and improve mental health outcomes is compelling and warrants a definitive study to determine effectiveness.

## Reference

## Figures and Tables

**Table 1:** Bootstrapped weighted descriptive statistics of the Canadian adult population.

Control Variables	Measure
average age (years)	45
average household income (Canadian dollars)	77,548
average weight (kg)	78
average height (cm)	169
males	50%
marital status: married	51%
education	-
secondary or less	30%
colleague/trades/certificates	45%
university or higher	25%
white racial Origin	82%
student	12%
smokes daily	17%
regular drinker	68%
used prescription drugs for recreational purposes	3%
used or tried street drugs	15%
weight status (as defined by the World Health Organization)	-
underweight	2%
normal weight	42%
overweight	35%
obese	20%

**Table 2:** Indicators of mental health.

Dependent Variables	% of Total Population
emotional problems (HUI3)	-
life not worthwhile	0.3%
very unhappy	0.7%
somewhat unhappy	3.1%
somewhat happy	17.5%
happy in life	78.4%
self-perceived mental health	-
poor	0.9%
fair	4.3%
good	21.9%
very good	38.8%
excellent	34.1%
self-perceived stress	-
extremely	3.2%
quite a bit	16.8%
a bit	44.0%
not very	26.4%
not at all	9.7%
self-perceived health	-
poor	2.5%
fair	8.8%
good	36.4%
very good	38.4%
excellent	13.8%

**Table 3:** Coefficients for the association of serum 25(OH)D concentrations (per 25 nmol/L increase) with mental health indicators in various regression models.

Dependent Variable	Unadjusted Model	+Demographics	+Socioeconomic	+Life Style	+Health
Emotional health	1.24 *** (0.06)	1.19 *** (0.06)	1.17 *** (0.06)	1.15 *** (0.05)	1.16 *** (0.06)
Mental Health	1.10 *** (0.04)	1.10 ** (0.04)	1.09 ** (0.04)	1.07 * (0.04)	1.08 * (0.04)
Stress	1.09 ** (0.05)	1.10 ** (0.04)	1.10 ** (0.05)	1.09 * (0.05)	1.10 ** (0.05)
General Health	1.23 *** (0.04)	1.20 *** (0.04)	1.19 *** (0.04)	1.16 *** (0.04)	1.17 *** (0.04)

*, **, *** indicates 1%, 5%, 10%, significance levels, respectively. Bootstrap standard errors in parentheses.
